# Reports on the Hospitals of the United Kingdom

**Published:** 1911-12-30

**Authors:** Henry Burdett


### REPORTS ON THE
### Hospitals of the United Kingdom.

By SIR HENRY BURDETT, K.C.B., K.C.V.O.

### LIX.—The Victoria Hospital for Sick Children, Hull.

This hospital, established in 1872, the present buildings having been erected so recently as 1891, though conducted with zeal and spirit by the present resident staff, caused us genuine disappointment. Those who are responsible for the present buildings had a great opportunity to provide Hull with one completely modern and up-to-date hospital; but they sadly omitted to avail themselves of it. The buildings are so planned as to create varying levels on several floors, entailing the introduction of steps or short staircases. These faults culminate in placing the operation theatre at the very top of the building, where it is perched like a chimney pot, to which the only access is by short flights of steep steps. Anything more opposed to the requirements of a modern hospital, and especially of a children's hospital, can hardly be conceived than this operation theatre with its attendant and unnecessary dangers of approach. Other regrettable features are the absence of adequate accommodation for the nursing staff and the grievous need for ventilation and thoughtful arrangement in the out-patient department, the atmosphere of which, when we inspected it, was well calculated to make healthy people ill, so that its effects upon sick patients who attend must be seriously detrimental.

### Will the Mothers of Hull Please Note?

We could wish that every tender-hearted mother in Hull could be brought suddenly into the centre of the out-patient hall of this hospital at the busiest time of the day, for then we are certain its present indefensibly bad condition and inadequacy would disappear with the rapidity of an electric shock. The present matron and her staff have our sympathy in the conditions under which they have to do some of their work. They give constant evidence of whole-hearted devotion to the welfare of the hospital and the care of its inmates, but it is the truth to say boldly that either the present out-patient department should be, closed at once, or entirely reconstructed upon hygienic principles, without a moment's avoidable delay. We never remember a graver instance of the grievous effects of ill-thought-out plans on the construction and arrangement of hospital buildings. Where the responsibility rests for the many faults which have been committed, we have no knowledge, but the fact remains that the sooner it is recognised and the speedier adequate remedies are applied the better.

### The General Arrangements.

We have said that the present matron, Miss D. Lyon, who was trained at the London Hospital and has taken exceptional and commendable measures to fit herself for the post she now occupies, is full of zeal and energy, and, in justice we may add, highly competent too. She deserves and should receive the whole-hearted support, not only of her committee but of the people of Hull, so as to make this necessary hospital as modern and up-to-date as possible. One great difficulty we should say which she has to face is that too many young women are employed on the nursing staff. The present system should be modified by the introduction of more fully-trained and qualified nurses. The employment of too many young women on the staff of a children's hospital often becomes, in practice, a cruelty to the patients, whilst its effects, on the general order and cleanliness of wards and offices, are readily apparent to the trained observer. Under such a system, for instance, sink traps are constantly liable to stoppage from an absence of care due to the youth and inexperience of the staff. All these evils are readily avoidable where the committee trusts a competent and highly trained matron and adopts her recommendations for reform.

### Very Creditable to the Management.

The wards and balconies form the best features of this hospital and are on the whole good. The latter must be of immense advantage to the little patients. It is creditable to the management and also to the staff that they should have established a convalescent home at Hornsea, for such accommodation, like the balconies, is essential to the adequate treatment of a type of cases unhappily far too frequent in Hull. The furniture and fittings of the wards, however, notably the cots and the lockers, need renewal, and we commend to the committee's attention the absence of certain modern appliances. One excellent fact about this hospital is the thorough way in which the domestic staff does its work. The condition of the crockery, pantries, and other matters under their control was highly creditable to all concerned. In the same way the excellent condition of the child inmates, who were evidently well cared for, was pleasant to witness. It speaks strongly in commendation of the standard of personal service which the present matron properly makes a special feature of her system of management. The honorary medical staff, though their difficulties in regard to operations and the administration of the out-patient department must prove grievous handicaps, evidently endeavour successfully to secure that the inmates of this hospital shall have the advantage of every modern treatment available. We commend the needs, requirements, and work of this hospital to the immediate attention and warm support of the people of Hull.

